# Patient‐Derived Melanoma Immune‐Tumoroids as a Platform for Precise High throughput Drug Screening

**DOI:** 10.1002/advs.202408707

**Published:** 2024-10-30

**Authors:** Juliana Viegas, Sofia Costa, Sofia Dias, Catarina Leite Pereira, Bruno Sarmento

**Affiliations:** ^1^ i3S–Instituto de Investigação e Inovação em Saúde Universidade do Porto Rua Alfredo Allen 208 Porto 4200‐135 Portugal; ^2^ INEB–Instituto de Engenharia Biomédica Universidade do Porto Rua Alfredo Allen 208 Porto 4200‐135 Portugal; ^3^ IUCS‐CESPU–Instituto Universitário de Ciências da Saúde Rua Central de Gandra 1317 Gandra 4585‐116 Portugal

**Keywords:** 3D models, in vitro model, multicellular spheroids, patient‐derived, skin cancer

## Abstract

In vitro models are crucial in cancer research, but they must truthfully mimic in vivo tumors for clinical relevance. The development of unprecedent melanoma quadruple multicellular tumoroids (MCTs) is proposed comprising tumor cells, keratinocytes, fibroblasts, and monocytes that replicate tumor architecture, tumor microenvironment, and secretome behavior. These MCTs of 300 µm in diameter secreted keratin and collagen, showing complexity proportional to their cell combinations. Further, closely resembled in vivo tumors in terms of cells organization, growth, progression, and immune behavior. Drug screening using these MCTs demonstrated their potential as patient‐derived platforms for precision medicine. These findings highlight the true value of MCTs for studying melanoma biology and testing therapeutic interventions with greater precision and relevance to human disease.

## Graphical abstract

Patient‐derived melanoma immune‐tumoroids are developed, consisting of tumor cells, keratinocytes, fibroblasts, and monocytes. These 300 µm MCTs replicate tumor architecture and behavior, closely resembling in vivo tumors. Their secretome panel demonstrate their immune reassembly capacity. MCTs demonstrate significant potential for drug screening and precision medicine, enhancing melanoma research and therapeutic testing.

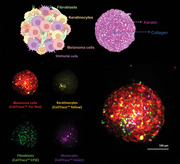

## 1 Introduction

Cutaneous melanoma represents a significant challenge in oncology due to its aggressive nature and propensity for metastasis. Despite advancements in treatments, the development of effective therapeutic strategies remains a pressing need.^[^ ^1^, ^2^ ^]^ One of the major drawbacks in the development of new treatments options is related to the way novel therapeutics approaches are tested, normally restrict to in vivo studies, that are very little translatable to human reality, delaying whole process.^[^ ^3^, ^4^ ^]^

In vitro models are invaluable tools in cancer research, offering a controlled environment for investigating tumor biology and drug responses in human cells, with less ethical concerns and limitations associated with animal experimentation. However, traditional in vitro models often fall short in mimicking the complexity of the tumor microenvironment (TME), which comprises not only malignant cells but also various stromal components and immune cells that are crucial in shaping tumor progression and response to therapy.^[^ ^5^ ^]^

By incorporating diverse cellular components and 3D architectural features resembling native tissues,^[^ ^1^, ^5^ ^]^ more complex in vitro platforms hold promise in enhancing our understanding of tumor‐host interactions in a patient‐specific scenario, and thus facilitating the development of targeted therapeutic interventions in terms of precise medicine.^[^ ^6^ ^]^ 3D models, such as tumoroids, play a pivotal role as alternative models compared to simplistic 2D cell culture systems and animal models, which often fail to fully recapitulate human pathophysiology.^[^ ^4^, ^7^ ^]^ Hence, the utilization of 3D tumoroids encompassing a heterogeneous mix of tumor cells, keratinocytes, fibroblasts, and immune cells emerges as a compelling approach to more accurately replicate the physiological conditions encountered in vivo.^[^ ^8^ ^]^

Herein we proposed the assembly of melanoma tumoroids with immune features as an innovative and high throughput in vitro platform for drug testing. By integrating stromal and immune cells within the tumoroid structure, this model not only mimics the intricate interplay between cancer cells and skin cells, but also provides a versatile platform for assessing the efficacy of therapeutic agents and combination therapies. Through systematic characterization of the tumoroid 3D structure, evaluation of drug responses and secretome, with and without treatment, in a physiologically relevant setting, we aim to accelerate the discovery and development of novel treatment strategies tailored to combat cutaneous melanoma and improve patient outcomes.

## 2 Results

### 2.1 Multicellular Tumoroids (MCTs) Growth and Metabolism Pattern

**Figure 1:**
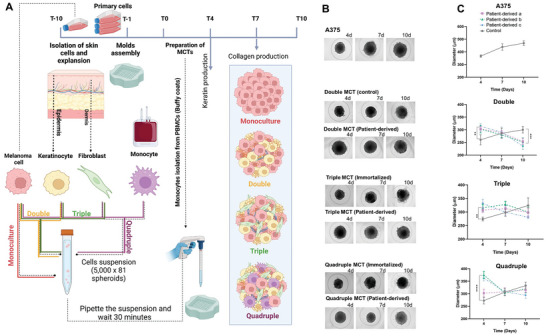
Standardize MCTs assembly protocol using microtissue molds since the isolation of cells, culturing and MCTs preparation and first steps of characterization. A) Keratinocytes and fibroblasts were isolated from human skin and cultured until confluence, while A375 cells were also expanded; At the day of MCTs production, the monocytes were isolated from buffy coats. The suspensions were prepared mixing the proper number of each cell and then, they were assembled into microtissue molds at time zero (T0). All conditions have 5,000 cells per spheroid, being the ratios 2:1 (A375 to keratinocytes) – Double, 2:1:1 (A375 to keratinocytes to fibroblasts) – Triple, and 2:1:1:1 (A375 to keratinocytes to fibroblasts to monocytes) ‐ Quadruples. Since there, B) the MCTs were monitored using Zoe microscope and the media was changed every two days. C) the diameters of MCTs were determined using ImageJ (NIH) software and OrganoSeg software. Data are represented as mean ± SD, *n* = 10. See also Figure S1 (Supporting Information) for the spheroid control without tumor cells.

The MCTs were successfully developed from immortalized cells as controls and from patient‐derived cells (**Figure** **1**A) in order to set up a robust model, showcasing the peculiarities regarding the use of patient‐derived material while highlighting the circumstances in which the use of immortalized cell lines can yield sufficient and trustable readouts.

All MCTs conditions – monoculture, double, triple and quadruple, formed compact, reproducible structures with consistent characteristics across microtissue molds. Initially, the A375 monocultures were less compact and exhibited more pronounced growth over time compared to the others MCTs conditions. The double and triple conditions were more compact than the monocultures, although they also presented size increase over time. The quadruple MCTs exhibited the slightest growth over time, displaying cell detachment from the periphery after the 7^th^ day of culture. In MCTs produced from patient‐derived cells (PD), all conditions – double, triple, and quadruple an initially less compact MCTs was shown when comparing to the immortalized ones. However, they became more compact over time and, by the 7^th^ day, their diameters were similar to those of the control conditions (immortalized cells) (Figure 1B,C) reaching at day 10 in culture up to 254 ± 12 µm, 324 ± 28 µm, and 332 ± 13 µm for double‐PD, triple‐PD and quadruple‐PD MCTs respectively.

**Figure 2:**
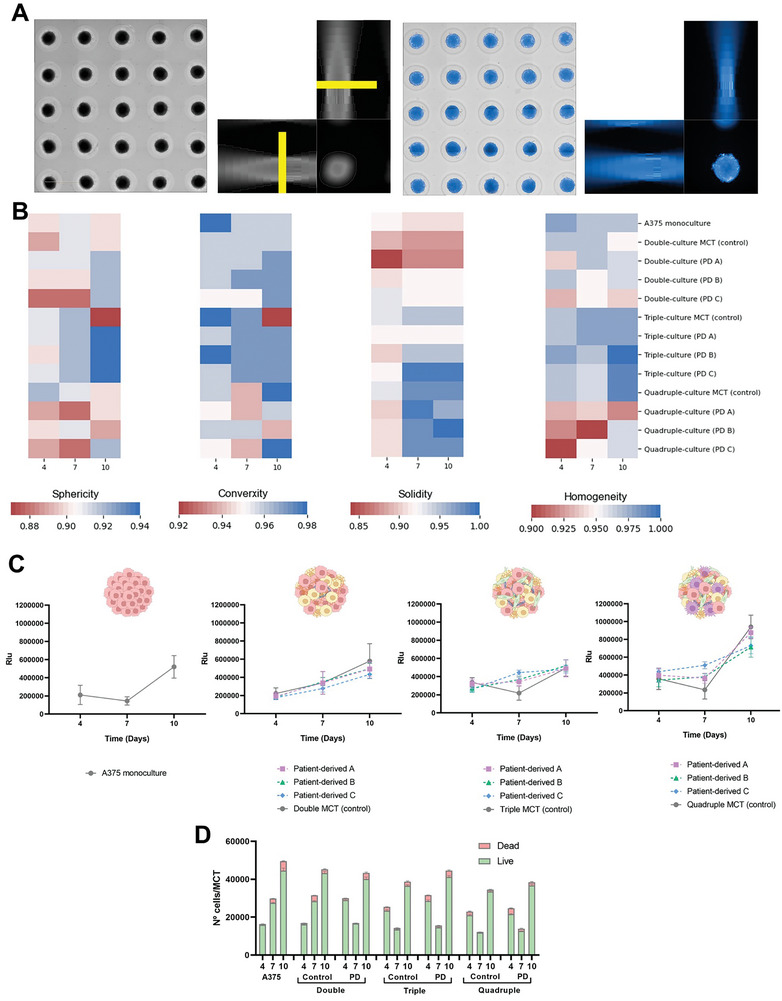
MCTs characterization by A) high‐throughput images collection adopting an in‐house protocol in the Operetta High Content Imaging System based on MCTs identification using Hoechst. Through those images was possible to determine crucial parameters such as B) sphericity, convexity, solidity and, homogeneity. Moreover, C) the metabolic activity was measured by ATP (CellTiter‐Glo) and D) the MCTs dissociation determine the number of cells in each MCTs and the number of them alive and dead. Data are represented as mean ± SD, *n *= 10.

Though high‐throughput imaging (**Figure** **2**A) was possible to efficiently follow‐up the spheroids growth and morphology inside the tissue micro molds. As expected, the monocultures were the most homogeneous while the triple and quadruple MCTs were the least homogeneous, exhibiting a higher number of sites with cells growing outward from the main spherical structure. This phenomenon also occurred, though less pronounced, in the mono and double conditions. At the end of the 10‐day cultivation period, the triple MCTs were the most spherical, the quadruple MCTs were more convex, and both were more solid compared to the monocultures and double MCTs (Figure 2B).

Regarding the metabolic activity (Figure 2C) of the MCTs, it was possible to observe that the metabolic activity of all MCTs types increased over time, particularly at day 10. This increase was particularly evident in the more complex MCTs – quadruple, surpassing all other conditions by ≈20%. Interestingly, the counted cells after dissociation (Figure 2D) showed a correlation between number of live cells and metabolic activity.

### 2.2 Histological Findings Suggest ECM Production and High Expression of a Melanoma Marker

**Figure 3:**
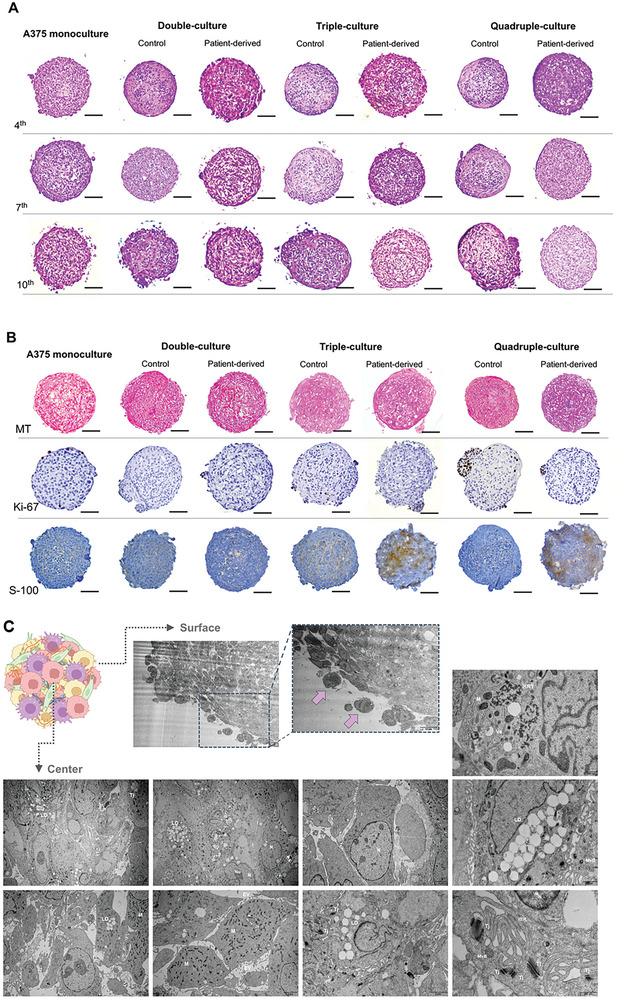
MCTs architecture and expression of extracellular matrix (ECM) and melanoma marker were assessed in the 7^th^ day of culture by A) hematoxylin‐Eosin histological analysis and B) Thricome Masson differential histology for keratin and collagen, immunohistochemistry for ki‐67 (DAB detection) and S‐100 protein (DAB detection). The scale bar indicates 100 µm in all images. Representative images of an entire 9x9 microtissue mold for each condition. C) Transmission electron microscopy (TEM) iMCTS (quadruple) ultrastructure imaging. Images showed cell‐cell interactions and accumulation of ECM. Images were obtained with 12, 15, and 35 k magnification. These are representative images and, they were collected since the border to the center crossing to the opposite border. N = nuclei, Nu = nucleolus, ER = endoplasmic reticulum, rER = rough endoplasmic reticulum, M = mithocondria, EV = extracellular vesicles, Ve = vesicles, MvB = multivesicular bodies, App = apoptotic podies, Ap = apoptosis, Nec = necrosis, ECM = extracellular matrix, Tj = tight junctions, KRT = Keratin, LD = Lipid droplets. See also Figure S2 (Supporting Information) for TEM images of monoculture, double and triple MCTs. Arrows showing monocytes.

Histological studies (**Figure** **3**A) can provide relevant insights into the architecture of the MCTs, allowing for a correlation between their organization, structural appearance, cells distribution and the ECM present in the structure. Herein, we have compared the histological features of patient derived MCTs with controls through hematoxylin‐Eosin (HE), Thricome Masson for keratin and collagen, and immunohistochemistry for ki‐67 (DAB detection) and S‐100 protein (DAB detection). HE staining show compact structures in all the MCTs with evident structural differences over time. However, at no time until the 10^th^ day was necrotic core observed in any condition. The tumoroids composed exclusively of tumor cells showed a uniform architecture, presenting no to low production of extracellular matrix (ECM) comparing with the heterotypic MCTs. With the addition of keratinocytes, it is evident that a higher ECM deposition occurred (Figure 3A), and the tumoroids become anisotropic. Also, though HE staining, it is also possible to notice that in the triple and quadruple MCTs there was a growth of cells migrating to the periphery, creating spots outside the main site.

Based on insights provided by the HE images, an in‐depth evaluation of the ECM and the differences conferred by this anisotropic structure was essential (Figure 3B). ECM production was confirmed to be minimal in the monoculture tumoroids, with keratin (red‐pink labeling) being the main ECM secreted by the double MCTs, both in immortalized and patient‐derived MCTs. Collagen deposition (blue labeling) is more evident in the triple and quadruple MCTs, as expected. However, in the quadruple MCTs, with the addition of immune cells, a significant increase in collagen production was observed, particularly in the center of the quadruple PD MCTs.

Proliferative capacity was also evaluated through the staining with ki‐67, a proliferative marker. Significant differences were observed among the MCTs, with the quadruples being the ones that displayed a higher number of proliferative cells. Interestingly, the quadruples MCTs also showed higher tissue expression of S‐100, a calcium‐binding protein that is often used as a biomarker for melanoma,^[^ ^9^ ^]^ followed by the triple MCTs. Importantly, the PD tumoroids displayed a high S‐100 staining, suggesting that the cellular source of the tumoroids can impact in the expression of certain markers, altering the model resemblance to in vivo melanoma tumors.

### 2.3 Ultrastructure Showing Differences Among MCTs and Immune‐MCT

In the pursuit of characterizing and observing the capacity of tumoroids to resemble in vivo melanoma tumors, it was determined that the day 7^th^ is the optimal time point for MCTs characterization and application in screening studies, and also that the patient‐derived MCTs were the ones with the greatest capacity to mimic the in vivo microenvironment of melanoma, compared to the immortalized cells. As such, the ultrastructure of patient‐derived MCTs was studied using transmission microscopy (Figure 3C; Figure S2, Supporting Information). The quadruple MCTs exhibited cells with a morphology compatible with those of cells that composed the model, tumoral cells with high mitochondrial activity, keratinocytes with keratin production evidenced, fibroblasts being the most elongated cells, and monocytes in the periphery. The quadruple MCT showed signs of high metabolic activity (massive quantities of mitochondria), corroborating previous data on the metabolic activity (Figure 2C). A high amount of ECM, evidenced by keratin and protein structures, suggest the deposition of collagen. A high detachment of cells on the surface is confirmed, as shown in Figure 3, that are compatible with monocyte morphology.^[^ ^10^ ^]^

Two features differentiate the ultrastructure of the quadruples from the other MCTs: i) the intense production of lipid droplets, as well as ii) an intense cell‐to‐cell communication evidenced by numerous T‐junctions.

### 2.4 Cells Distribution through the 3D Structure

**Figure 4:**
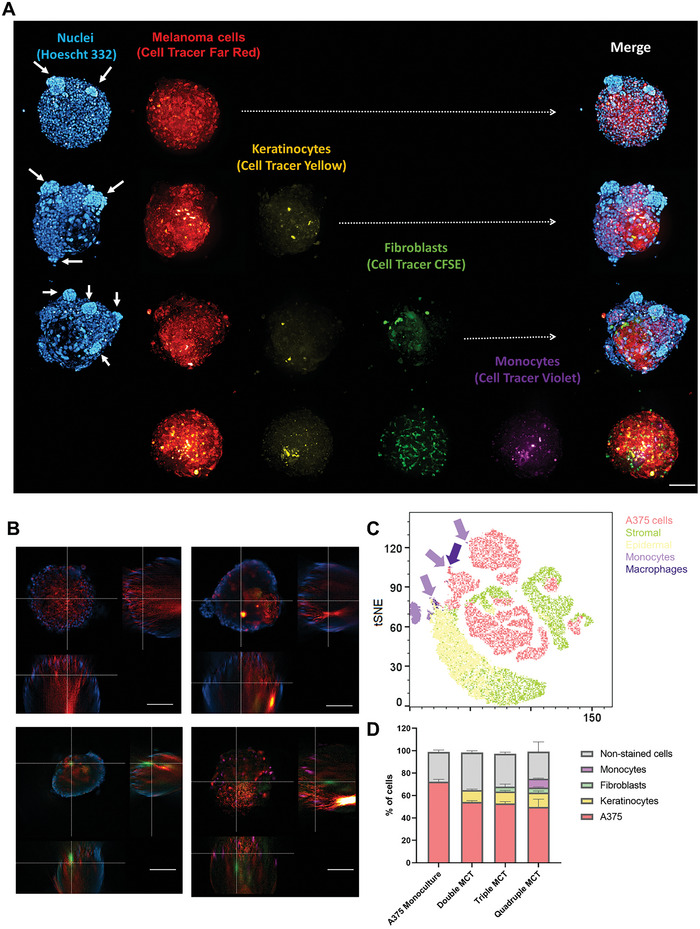
High‐throughput imaging of MCTs using CellTrace to track each cell, being A375 cells labeled with CellTrace Far Red (Invitrogen, C34572; 630/661 nm), the keratinocytes labeled with CellTrace™ Yellow (Invitrogen, C34573; 532/561 nm), the fibroblasts labeled with Vybrant CFDA SE CellTrace and, the monocytes labeled with CellTrace Violet (Invitrogen, C34557, 405⁄450 nm). The nuclei were stained with Hoechst 33342 with exception of quadruple condition. The imaging acquisition was made using (A) PerkinElmer Opera Phenix Microscope and B) THUNDER Imaging Systems (Leica). The same MCTs were dissociated and the CellTrace was used to identify each single cells by Flow Cytometry (FACSCanto II, BD Biosciences, USA) and the data was plotted as a C) tSNE graph of the concatenated samples after exclusion of debris and doublets. D) MCTs had also their population quantified using the Flow Cytometry data, however since the MCTs were kept in culture 7 days to perform these experiments, some cells duplicated and had lost the CellTrace, thus in the graph those cells were represented as non‐stained cells. Bars represent 100 µm.

To determine the cellular organization and their distribution over time, the MCTs were evaluated by fluorescence microscopy at the 7^th^ day in culture, where each cell composing the MCTs was previously labeled with different CellTrace, enabling to distinguish each cell type with a different color. **Figure** **4** shows the spatial distribution of the cells. As expected, the majority of cells that compose the MCTs are the tumor cells, that became clustered with the addition of other cell types. An explanation for this can be due to the ECM produced, creating an anisotropic distribution in the structure. It is noted that the distribution of keratinocytes follows that of fibroblasts, which become morphologically different in the quadruple condition, suggesting differentiation.^[^ ^11^ ^]^ Another fact about fibroblasts is that they are highly capable of clustering with A375 cells, potentially indicating a differentiation toward cancer‐associated fibroblasts (CAFs).^[^ ^11^ ^]^ Monocytes are widely distributed on the periphery of the spheroid, with a minority located centrally. From the tSNE graph (Figure 4C), the macrophage population is positioned, in a bidimensional manner, close to the tumor cells, suggesting some interaction and potentially differentiation in tumor‐associated macrophages (TAMs).^[^ ^12^, ^13^ ^]^

**Figure 5:**
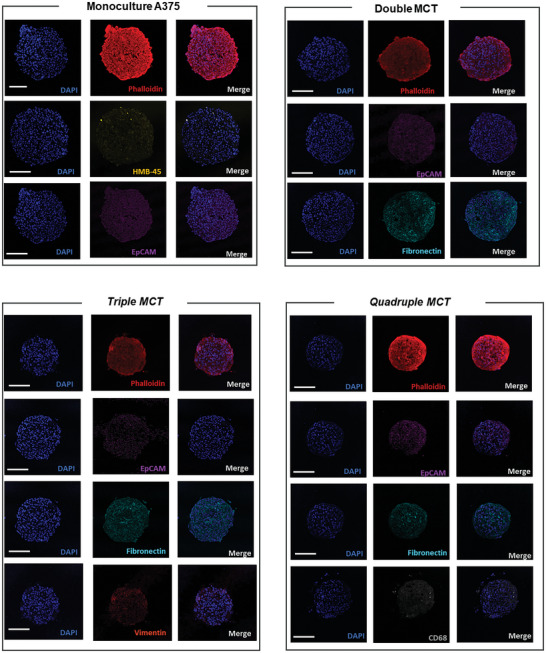
Immunofluorescence images obtained by Confocal. The A375 monoculture spheroid was analyzed for EPCAM and HMB‐45, the MCT double was analyzed for EPCAM and fibronectin, the MCT triple was analyzed for EPCAM, fibronectin and vimentin, and finally the iMCTs (quadruple) was analyzed for EPCAM, fibronectin and CD68. All MCTs were stained with DAPI and Phalloidin (Alexa Fluor 546). The slices were 3 µm of thickness, and immunoassay was independent. These are representative images of ten MCTs for each condition. Bars represent 100 µm.

Immunofluorescence images (**Figure** **5**) evidence the clustering of fibroblasts through vimentin, show the organization of monocytes (CD68), especially on the surface, and also confirm the intense production of ECM, indicated by the strong expression of fibronectin.

### 2.5 Immune Population and Secretome Characterization Resembling Immune Behavior

**Figure 6:**
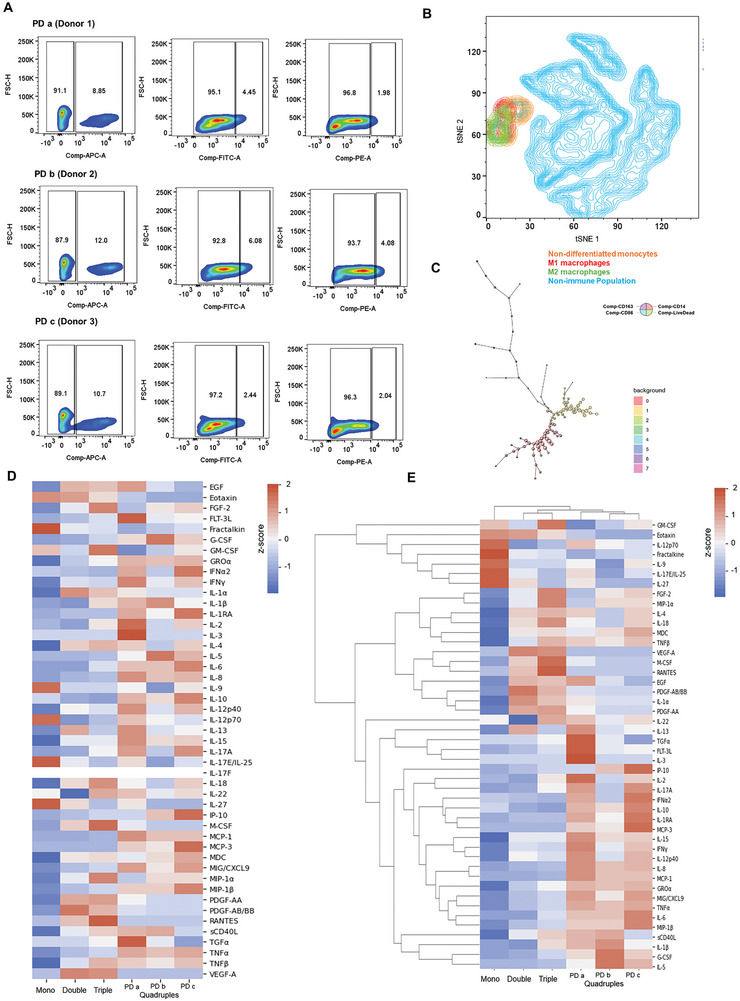
Immune population characterization by A) Flow Cytometry using specific Cluster of Differentiation for monocytes/ macrophages – First gate (CD14 – APC) and for differentiated M1 macrophages – Second gate (CD86 – FITC) and M2 macrophages – third gate (CD163 – PE). In all samples were included cell viability marker, Live Dead APC‐Cy7. B) tSNE graph generated from concatenated data from three different donors and C) their clustering generated by Flow‐SOM (FlowJo software). The immune behavior of MCTs were also characterized by cytokines secretion through multiplex Human Cytokine Panel A 48‐Plex Discovery Assay^®^. The data were analyzed using Python 3.9 with Pandas package for pre‐processing data and Seaborn for generating the D) heatmaps and E) Clustermaps. The plots were created from normalized data as z‐score, average of *n* = 3 for each condition.

Characterizing the immune population and determining the capacity of the immune‐tumoroids (quadruple MCTs) to secrete cytokines and chemokines, associated with tumor growth, resistance, invasion capabilities, and metastasis, is essential for understanding the biomimetic potential of MCTs as a model, inferring whether these 3D structures can provide an accurate representation of the in vivo microenvironment, that can be applied for therapy screening studies or even studies related to understanding the disease, molecular pathways, development, cell death, etc. In our final model, the quadruple MCTs percentage of monocytes that spontaneously differentiated into M1 and M2 macrophages was determined by flow cytometry (**Figure** **6**). About 10.5 ± 1.3% of quadruples MCTs population are CD14 positive, that includes monocytes and macrophages. Among those, 4.3 ± 1.5% are CD86 positive, being M1‐like phenotype macrophages, and 2.7 ± 0.9% are CD163 positive, being M2‐like phenotype macrophages.

When looking at the secretome of the MCTs, the quadruple MCTs displayed a cytokines profile characterized by the predominant secretion of TNF‐α, TGF‐α, MIP‐1β, MIG, EGF, MCP‐3, MCP‐1, IP‐10, IL‐17A, IL‐15, G‐CSF, INF‐α2, INF‐γ, IL‐3, IL‐2, IL‐13, IL‐12p40, IL‐10, IL‐8, and IL‐5. This pattern of secretion corroborates to characterize the quadruple MCTs as presenting an extremely high proliferative behavior, invasiveness and migration capability, while minimal signals of cell death, high treatment resistance and high complexity, compared to the others MCTs was observed (Table S1, Supporting Information). Through Clustermaps (Figure 6E), it was evidenced by increasing the cellular complexity of these models from mono to quadruple MCTs, a higher cytokine secretion was observed, suggesting that cell‐cell interaction is fundamental ro recreate the tumor microenvironment.

### 2.6 Screening of Therapies Highlighting Vemurafenib

**Figure 7:**
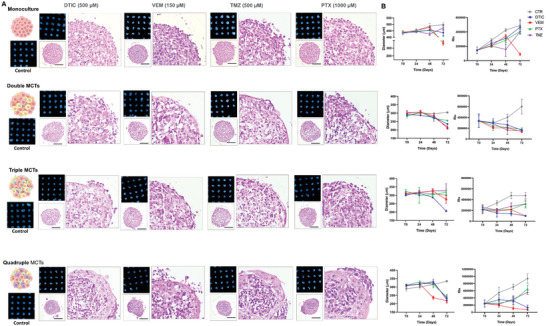
High‐throughput analysis of spheroids after treatments by Operetta High Content Imaging System which allow the A) follow‐up of microtissue molds during 72 h of treatment. After 72 h of treatments the MCTs were also characterized by tissue architecture by HE staining. B) Their diameters and metabolic activity during treatment were determined by measures of 5 x 5 matrix (at least 15 spheroids for each condition) – 10x magnification, and by CellTiter‐Glo 3D cell viability assay reagent respectively. The treatments were dacarbazine (DTIC) at 500 µm, Vemurafenib (VEM) at 150 µm, Paclitaxel (PTX) at 1000 µm and Temozolomide (TMZ) at 500 µm. The drugs concentrations were pre‐determined among four, and stablished as the optimal for all MCTs. Data are represented as mean ± SEM, *n* = 15. The scale bar indicates 100 µm in all images.

To validate the MCTs as efficient and high‐throughput platforms for therapy screening, four drugs were selected (**Figure** **7**) namely: dacarbazine (DTIC) at 500 µm and vemurafenib (VEM) at 150 µm, which are commonly prescribed for melanoma treatment, paclitaxel (PTX) at 1000 µm chosen to include a general drug for solid tumors, and temozolomide (TMZ) at 500 µm selected to represent drugs of choice in cases of metastases. The dosages of each drug were determined based on previous experiments (Figure S3, Supporting Information).

Given the known resistance of A375 cells to DTIC, it was expected that this drug would not impact the monoculture tumoroids.^[^ ^14^ ^]^ Interestingly, DTIC was able to reduce the diameter and metabolic activity of the others MCTs, especially the triples and quadruples, being ≈32 and 28% of decrease in relative diameter respectively. A high number of apoptotic cells were observed detaching from the main structure (Figure 7A). PTX and TMZ showed to have moderate actions, displaying control over MCT growth and causing cell death within the MCTs, exhibiting pyknotic nuclei and some signs of necrosis, but no remarkable action. VEM was the only drug capable of acting efficiently on all MCTs within 72 h, with significant effects on the quadruples already observed at 48 h. All combinations of MCTs, after 72 h of treatment with VEM, showed disaggregation, apoptotic cells on the surface, and pyknotic nuclei throughout the structure.

### 2.7 Impact of Drugs Over in the MCTs Secretome

**Figure 8:**
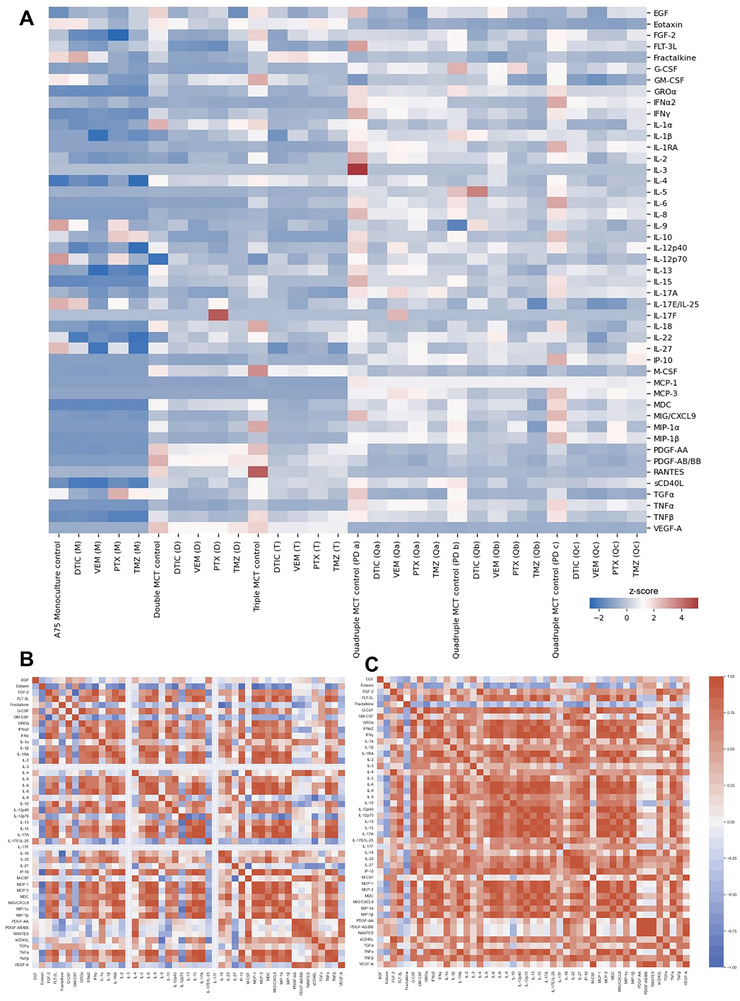
Cytokines panel (Human Cytokine Panel A 48‐Plex Discovery Assay) Heatmaps of MCTs controls and after each treatment with the drugs dacarbazine (DTIC) at 500 µm, Vemurafenib (VEM) at 150 µm, Paclitaxel (PTX) at 1000 µm and Temozolomide (TMZ) at 500 µm. The drugs concentrations were pre‐determined among four, and stablished as the optimal for all MCTs. A) General Heatmap and Correlation Heatmap of B) DTIC and (C) VEM. The plots were created from normalized data as z‐score.

To understand not only the impact of the drugs on tumor growth and progression, we have further looked into their secretome profile right after 72 h of treatment (**Figure** **8**; Figures S4 and S5, Supporting Information). DTIC suppressed more efficiently pro‐inflammatory cytokines (IL‐17A, IL‐8) and immune‐modulatory cytokines (INF‐α2, IL‐13) that promote tumor growth or immune evasion. At the same time, DTIC increased IL‐5, which may indicate a shift toward a more activated immune response, potentially enhancing anti‐tumor immune mechanisms.^[^ ^15^ ^]^ VEM decreased TNF‐α, INF‐γ, IL‐3, IL‐2, IL‐13, and IL‐12p40. These cytokines are involved in inflammation, immune activation, and tumor growth promotion. Thus, the ability to reduce these cytokines may indicate modulation of inflammatory responses and immune evasion pathways. PTX was able to decrease the levels of MIP‐1β, IP‐10, IL‐15, G‐CSF, IL‐3, IL‐13, and IL‐8. In general, these cytokines play roles in immune cell recruitment, inflammation, and tumor cell survival. This action might suggest modulation of the tumor microenvironment to reduce inflammation and inhibit pathways that support tumor survival and growth. Finally, TMZ reduced IL‐13, IL‐3, EGF, IP‐10, and IL‐15, which are cytokines related to immune modulation, cell survival, and growth factor signaling. The reduction of these cytokines may indicate suppression of pathways that promote tumor cell survival, proliferation, and immune evasion.

## 3 Discussion

The 3D models represent a significant advancement in the study of cancer biology, offering numerous benefits over traditional 2D cultures, enabling researchers to address key mechanistic aspects related to tumor development, progression, and response to treatments, providing a more comprehensive understanding of the disease. These models offer a controlled environment while simultaneously allowing the incorporation of patient‐derived cells, thereby capturing individual peculiarities and ensuring a high level of precision and relevance in the findings. Moreover, 3D models generate more humane readouts compared to in vivo studies, being easily translatable since they mimic the complex interactions within the tumor microenvironment. Also, following directrices of reducing animal experimentation, the use of 3D models avoids ethical issues related to animal testing, promoting a refinement approach to cancer research while still delivering robust and reliable data.

The MCTs proposed in this work aimed recapitulate the 3D complexity of an in vivo melanoma tumor, achieving three critical points: architecture and cross‐talk among cells, growth, and progression, and behavior especially immune behavior.

Using techniques that allow high‐throughput assembly, the MCTs were produced and extensively characterized, demonstrating high reproducibility, accuracy, and precision. This indicates that the preparation methodology is robust enough, even when using patient‐derived cells. This preparation method, adopting microtissue molds, has been utilized in several studies in the literature.^[^ ^16^, ^17^, ^18^ ^]^ Furthermore, in this study, high‐throughput imaging showcased the ability to produce these tumoroids in a highly standardized and rapid manner, while also efficiently characterizing and testing them.

The MCTs presented structures similar to in vivo tumors, nodular and anisotropic (except the monoculture), with well‐established cell‐to‐cell communication and extracellular matrix (ECM) production, notably keratin and collagen. They showed increased metabolism over time, culminating in growth and the appearance of several growth sites that may indicate potential sites for migration.

As postulated in the literature, the average diameter and uniformity are critical parameters to consider in the production of spheroids, since they are essential for achieving reproducible experimental results and may affect cell behavior and function as well as drug penetration and transport. The MCTs developed in this work, under all conditions, exhibited an average diameter of 300 µm. This relative diameter is considered suitable for drug permeability testing, allowing for comparisons between different drugs and between MCTs conditions. Azizipour et al. (2022) mentioned that to accurately evaluate drug efficacy on spheroids, the generation of uniform‐sized and compact spheroids is essential for comparing responses under various drug concentrations.^[^ ^19^ ^]^ Additionally, regarding the composition of the MCTs, we proposed maintaining a constant final number of cells per spheroid, only altering the ratio between the added cells. This is also considered a critical parameter for the evaluation of drug efficacy and compare results between different MCTs.^[^ ^20^ ^]^

Directly related to spheroid architecture, but also correlated with compactness and molecular permeability, ECM production is a key factor in spheroid production. The production and regulation of ECM are highly correlated with the model's ability to mimic in vivo tumor conditions. The MCTs in this study, particularly the triple and quadruple MCTs, demonstrated a high capacity for ECM production, specifically keratin and collagen. Keratin is known for almost 3 decades,^[^ ^21^ ^]^ as a marker for invasiveness and metastatic potential in melanoma. Its expression is well described and commonly observed in melanoma lesions in more advanced stages.^[^ ^22^ ^]^ On the other hand, collagen has a dual role in melanoma, it can control the progression by controlling the melanoma phenotype, driving to a controlled cell invasion and, at the same time, the type IV collagen can help in melanoma cells migration and might promotes mutations.^[^ ^23^, ^24^, ^25^, ^26^ ^]^

The monoculture tumoroids, MCTs produced only from tumor cells, resembled published works on melanoma spheroids composed by only tumor cells.^[^ ^7^, ^27^, ^28^, ^29^, ^30^ ^]^ These monoculture spheroids were generally less complex, with predictable growth and metabolism, minimal ECM production, and a cytokine secretion pattern considered typical for a tumor model.

When keratinocytes were added to create the double MCTs, a clear change in behavior was observed. The double MCTs became more compact, with increased keratin production and enhanced metabolism. These tumoroids were less homogeneous in cells distribution. Regarding cytokine secretion, double MCTs had significantly higher production compared to the monoculture, presenting an inflammatory proliferative behavior characterized by VEGF‐A, PDGF‐AB/BB, EGF, RANTES, and IL‐1α.

Adding fibroblasts, to form the triple MCTs, intensified differences were observed across all aspects – structure, ECM, and behavior. Structurally, the triple MCTs became more anisotropic, with cluster of fibroblasts involving tumoral cells. Despite that, they were more spherical, solid, and convex. They exhibited a higher number of growth sites outside the main spheroid, suggesting subpopulations of tumoral cells with metastatic potential, which is important to resemble melanoma lesions, since within stages, the cells become to migrate into the skin, changing their behavior, and consequently creating first, local metastasis and later on, migrating to distant tissues.^[^ ^31^, ^32^ ^]^

The interaction between tumoral cells, keratinocytes, and fibroblasts resulted in intense ECM production. The cytokine secretion pattern became more complex, indicating a high capacity for angiogenesis and inflammation.

Klicks et al. (2019) developed a mono‐, double‐ and triple‐cultures melanoma spheroids, being melanoma cells (SK‐MEL 28), keratinocytes (HaCaT), and fibroblasts (CCD‐1137Sk). They seeded the fibroblasts first, and added the keratinocytes and melanoma cells in the 3^rd^ day. The authors observed a distinguished organization of cells, when the tumoral cells stay in the borders of the spheroids. Thus, this organization was used to observe the migration of SK‐MEL 28 cells toward fibroblasts core.^[^ ^33^ ^]^ Schafer et al. (2022) developed a co‐culture melanoma spheroid with the same composition of cells than the work mentioned before, to study the differences in transfection comparing 2D and 3D. They concluded that the interactions between cells create a 3D structure much less permeable than the cells in single cultures and 2D.^[^ ^34^ ^]^

Grujic et al. (2023) studied the impact of mast cells on melanoma cells growth in 3D. Their findings suggested that occurred a phenotypic change in the melanoma cells in the presence of those cells, and with their media. Their results showed an effect on metabolic process, catalytic activity, AA transmembrane transporter activity, biosynthesis of amino acids, among others.^[^ ^33^ ^]^

Interestingly, in our work, the quadruple MCTs, with the addition of monocytes, stood out not only for their ability to organizationally mimic in vivo melanoma tumors but also for being highly metabolically active. The quadruple MCTs were less spherical, presenting a certain heterogeneity, as well as, observed in melanoma lesions in advanced stages.^[^ ^32^ ^]^ Also, the quadruple MCTs produced significantly more ECM compared to the other MCTs and were markedly more positive for the S‐100 protein, especially in the patient‐derived condition.

The histology findings of the quadruple MCTs closely resembled that of amelanotic superficial spreading cutaneous melanoma biopsies. Key features, such as the presence of pagetoid cells and clusters of atypical cells, commonly referred to as “buckshot scatter” in the epidermis, were observed at the 3D structure. Additionally, the tumoral cells displayed abundant cytoplasm, nuclear pleomorphism, and prominent nucleoli, hyperchromatic tumor cell nuclei, lesion embedded within the extracellular matrix (ECM) which match with melanoma histopathology.^[^ ^35^, ^36^, ^37^, ^38^ ^]^

In regard of ultrastructure, the quadruple MCTs started to exhibit a significant level of lipid droplets that was not observed for the others MCTs conditions. In melanoma, the tumor microenvironment is well balanced by melanocytic state, and the lipid droplets are correlate to the regulation os this melanocytic state.^[^ ^39^ ^]^ These cells, in melanocytic invasive state, are more invasive and have higher metastatic potential, often exhibiting resistance to therapies and consequently poor patient outcomes.^[^ ^40^ ^]^ Lumaquin‐Yin et al. (2023) concluded that melanoma cells in the melanocytic state exhibit increased fatty acid uptake and a higher number of lipid droplets, which are essential for maintaining fatty acid oxidative metabolism. A disruption of lipid droplet formation results in impaired cell cycle progression and reduced tumor growth. This indicates that targeting lipid droplet biogenesis could be a potential therapeutic strategy for melanoma, as the dependency on lipid droplets represents a metabolic weakness in these cancer cells.^[^ ^39^ ^]^ In this way, our results regarding the quadruple MCTs strongly suggest that the melanoma cells which composed it were in melanocytic state and they, consequently, presented higher number of lipid droplets that the other MCTs.

Still regarding ultrastructure, the quadruple MCTs also exhibited a high number of extracellular vesicles (EVs) and T‐junctions. The EVs are known to be organelles that mediate the crosstalk between melanoma cells and the cells that compose the tumor, mainly stromal and immune cells.^[^ ^41^ ^]^ Furthermore, the role of T‐junctions in melanoma is not completely understood. However, they might indicate that the cells which presented downregulated to occludin are starting a progression stage, but no significant correlation was observed in clinicopathological studies.^[^ ^42^ ^]^ Thus, our quadruple MCTs have clearly a high cell‐to‐cell communication, justifying all changes in structure and behavior, evidenced by the interplay between all cells.

In addition, they had a secretome consistent with the tumor scenario.^[^ ^43^ ^]^ Their cytokine profile indicated a high capacity for migration (EGF), proliferation, intense immune modulation (IL‐13, IL‐12p40, IL‐5, and IL‐10) and activation (MIP‐1 β, MIG, MCP‐3, MCP‐1, IP‐10, IL‐17A, IFN‐α 2, and IFN‐γ) as well as signs of tumor resistance behavior (IL‐5, IL‐8).

Finally, we investigated the immune population that the quadruple MCTs presented in the 7^th^ day in culture. The distinctive cell types including immune cells, can create a complex TME, ECM, present subpopulations, and many others characteristics that have tremendous impact in cancer progression and response to treatments. Afterward, the evaluation of immune cells of quadruple MCTs have corresponded to a melanoma lesion, resembling closely an in vivo tumor, especially regarding proportion of cells composing the MCT, and also M1‐ and M2‐ like macrophages phenotype.^[^ ^44^, ^45^ ^]^ The proportion of M1 and M2 phenotypes in melanoma can be easily switched by the TME and tumor secretome, but usually M1‐like are more inclined to inflammatory processes, and presented in the first stages, and M2‐like are related to invasiveness, increasing in advanced stages. However, several works have been trying to understand better the role of M1‐ and M2‐ like phenotypes in melanoma, and the works are not in accordance about M1; it is just clear that they are in high numbers at initial stages.^[^ ^12^, ^44^, ^45^, ^46^, ^47^ ^]^

Despite our choice to include with tumoral cells – the keratinocytes, fibroblasts, and monocytes, the inclusion of other kind of cells can help to mimic different stages of the disease. The addition of endothelial cells, for instance, could benefit the resemble of more advanced stages which the presence of angiogenic potential is more evident, allowing then, drug studies targeting this pathway.^[^ ^17^ ^]^ Related to immune behavior, the monocytes could be replaced for instance for T‐cells, open possibilities to study immunotherapies targeting tumor‐infiltrating lymphocytes (TILs). However, the inclusion of T‐cells has to be done carefully, to avoid immune reactions especially heterologous reaction. Herein, we proposed the inclusion of monocytes since these immune cells are also extremely important in the cancer context and are commonly found in biopsies of cutaneous melanoma patients (≈10% of cells population). The M1 and M2 phenotypes can help to define the prognosis, since when the number of M2 – Tumor‐associated macrophages (TAMs) are linked to advanced stage, angiogenic, and metastatic behavior. Thus, the inclusion of an immune cell like monocytes opens up future opportunities for using the model to target TAMs, a strategy currently being explored as a potential therapeutic approach in cancer treatment.^[^ ^48^, ^49^, ^50^, ^51^ ^]^

The secretome analysis of the MCTs showed that the monoculture tumoroids are the simplest and least complex. The double MCTs secrete cytokines associated with angiogenesis, cell proliferation, and survival, aligning with literature on the relationship between keratinocytes and melanoma cells. The triple MCTs are much more complex than the mono‐ and double cultures, exhibiting strong secretion of cytokines and chemokines related to angiogenesis (such as VEGF‐A and PDGF‐AA), inflammatory markers (such as TNF, IL‐22, and IL‐4), and indicators that fibroblasts are active, such as FGF‐2. Additionally, the triple MCTs' secretome included cytokines involved in immune recruitment and activation, such as MIP‐1α, M‐CSF, and GM‐CSF;^[^ ^12^ ^]^ The quadruple MCTs are the most complex, secreting numerous cytokines and chemokines indicative of extremely high proliferation rates, invasiveness, migration, and suggesting a profile likely to result in drug resistance.^[^ ^15^ ^]^

After extensive characterization, the MCTs were subjected to high‐throughput drug screening with drugs indicated for the treatment of melanoma at different stages. Dacarbazine (DTIC) is the chemotherapy drug indicated for melanoma, particularly in stages where surgical options are not viable and there are indications of migration or metastatic processes.^[^ ^52^ ^]^ Vemurafenib (VEM) is an oral medication used in the treatment of cutaneous melanoma, specifically for patients whose tumors exhibit a mutation in the BRAF gene (most commonly the BRAF V600E mutation).^[^ ^53^ ^]^ To include a more general chemotherapeutic agent, Paclitaxel (PTX) was selected for its action in melanoma,^[^ ^54^ ^]^ especially in combination with other drugs. For cases of brain metastasis, temozolomide (TMZ) was chosen as it is one of the most common drugs used.^[^ ^14^ ^]^

The structural changes, which likely altered the permeability of the MCTs, along with behavioral changes in terms of growth, metabolism, and cytokine secretion, had a significant impact on the outcomes of the evaluated therapies. In the clinical scenario, it is expected that DTIC and TMZ cause DNA damage, apoptosis, and necrosis to the tumor, as well as the literature indicated that using DTIC the clinicians had achieved a more controlled growth pattern of the tumor lesion but the combined therapy can be a more effective approach.^[^ ^55^, ^56^ ^]^ For VEM is expected to have an increasing in necrosis followed by a decreasing in metabolic activity. In the clinic, VEM is also correlated to inducing cells senescence, tumor size reduction, and some histologic findings of incomplete apoptosis.^[^ ^57^ ^]^ Finally, for PTX, the literature pointed this drug as a moderate efficacy based on apoptosis, evidencing abnormal mitotic figures.^[^ ^58^, ^59^ ^]^ Our results pointed that we achieved comparable results with the clinical histopathology observation, which PTX indeed controlled the growth with no remarkable effect; DTIC was able to reduce the diameter and induce apoptosis, also controlling the growth; TMZ caused evident necrosis in the whole structure likewise VEM, which caused also cells disaggregation and intense apoptosis.

In the monoculture spheroid, only VEM was able to reduce its metabolism, which is expected given that the A375 cell line ports the V600E mutation.^[^ ^60^ ^]^ For the double MCTs, a growth control effect was observed, where all tested drugs were able to prevent the MCT from continuing to grow compared to the untreated control MCT. In the triple MCTs, only DTIC and VEM had a pronounced effect in reducing metabolism. The same was observed in the quadruple MCTs, DTIC, and VEM significantly reducing metabolism. However, in terms of diameter, all drugs were effective in the quadruples. This result suggests that PTX and TMZ increased cell death in peripheral cells but did not reduce the metabolism of the remaining cells. This pattern is confirmed by observing the cytokines secreted after 72 h of drug exposure, where indicators of cell death were minimal compared to the secretion of cytokines indicating tumor survival.

Overall, related to proliferation, both DTIC, VEM, and TMZ targeted pathways involved in cell growth and survival. Highlighting DTIC effect in increasing IL‐5, that suggests a favorable shift in immune response and TME modulation. Related to invasiveness, PTX and VEM showed significant reductions in cytokines that promote invasion and metastasis, indicating strong potential in controlling invasive behavior.^[^ ^61^ ^]^

Ultimately, after extensive characterization and validation of the MCTs, it is important to highlight that the proposed model is not only highly reproducible but also easily translatable to clinical settings due to the simplicity of its methodology. The tumoroids were generated using a straightforward approach, making it accessible for use in routine clinical workflows. In histopathology laboratories, where patient samples are collected and analyzed, a portion of the tissue could be set aside for cell isolation and the assembly of MCTs prior to fixation. Since this is a patient‐specific approach, there is no need for cell expansion, allowing the MCTs to be isolated and produced on the same day. Within just seven days, clinicians could have a personalized tool at their disposal to test different treatments and observe patient‐specific responses. Additionally, smaller healthcare centers could establish collaborations with universities or other diagnostic laboratories to carry out this process, which can be easily implemented in any lab with basic cell culture infrastructure.

## 4 Conclusion

In this study, we proposed a melanoma multicellular tumoroid (MCT) model that successfully recapitulates the 3D complexity of an in vivo melanoma tumor, achieving three critical points: architecture, behavior, and development. Our results demonstrated that the MCTs closely mimic the structural architecture of melanoma tumor, with distinct cellular organization and extracellular matrix (ECM) production, particularly when incorporating multiple cell types such as keratinocytes, fibroblasts, and monocytes. The MCTs exhibited key features of melanoma, including anisotropy, high metabolic activity, sites of growing generating “new tumors”, which in vivo will generate local metastasis, several histopathological findings, secretome patterns, and responses to chemotherapeutic agents, reflecting the dynamic interactions and resistance mechanisms observed in clinical scenarios. Furthermore, the development of these MCTs showed significant resemblance to tumor growth and progression, with observable differentiation of immune cells and the formation of tumor microenvironment (TME) similar to those in patient‐derived tumors in vivo. These findings highlight the potential of our model as a valuable tool for studying melanoma biology and testing therapeutic interventions with greater precision and relevance to human disease.

## 5 Experimental Section

### Material

Keratinocytes‐SFM (Thermo Fisher Scientific); Dulbecco's Modified Eagle Medium (Biowest); Penicillin‐streptomycin (Gibco); RosetteSep (Stemcell Technologies); Fibroblasts medium (Innoprot); Histopaque‐1077 (Sigma); Microtissue molds 3D Petri Dish (Merk); Formaldehyde 20% Solution (Electron Microscopy Sciences (EMS)); Trypan Blue 0.4% solution (Gibco); CellTiter‐Glo (Promega); Gill's Hematoxylin solution (Lusoplex); Alcoholic eosin (Lusoplex); Entellan mounting medium (Merck); Masson Trichrome (Epredia, Thermo Fisher Scientific); Histogel (Epredia, Thermo Fisher Scientific); All CellTrace (Invitrogen); Hoechst 33 342 (MedChemExpress); Accutase (Gibco); Versene (Gibco); 4′,6‐diamidino‐2‐phenylindole (DAPI) (Sigma‐Aldrich); Alexa Fluor 546 Phalloidin (Sigma‐Aldrich); Dacarbazine (MedChemExpress); Vemurafenib (MedChemExpress); Paclitaxel (Ruixibiotech); Temozolomide (Ruixibiotech). All antibodies are listed in the Table S2 (Supporting Information).

### Ethics Statement

The primary skin cells, keratinocytes, and fibroblasts were isolated from foreign skin obtained after abdominoplastic surgery performed at Department of Plastic Surgery of Centro Hospitalar Universitário São João (CHUSJ) (Protocol 90/17). The monocytes were isolated from surplus buffy coats from healthy blood donors provided by the Department of Immuno‐hemotherapy of CHUSJ (Protocol 90/19). Procedures were in accordance with the ethical approvement by the CHUSJ Ethics Committee.

### Cells Isolation and Culturing

The human skin was collected in Centro Hospitalar Universitário de São João (CHUSJ) after plastic surgery and the specimen was directed to i3S in a sterile flask. The skin fragment was kept in PBS with 5% (v/v) of penicillin‐streptomycin (pen‐strep) at 4 °C during 4–6 h (time‐dependent to size). In a flow chamber, the skin fragment was washed twice with sterile cold PBS, the subcutaneous tissue was completely removed, and the deep dermis was cleaned and mechanically separated from epidermis. In two different tubes, the epidermis and dermis in small fragments (< 2 mm) was incubated at 37 °C with 5 mL of trypsin during 2 h. After that, a centrifugation step (2000 rpm, 7 min) pelleted the tissue, and two steps of washing with PBS at 37 °C proceeded. Using a mesh, the pellet resuspended in complete media was filtered and then the primary keratinocytes and fibroblasts were cultivated. The primary keratinocytes were cultivated in keratinocytes‐SFM supplemented with 2.5 µg of EGF human recombinant, 25 mg of bovine pituitary extract until reach ≈90% of confluence. Purification step of keratinocytes regarding other cells such as endothelial and melanocytes, was performed by trypsin in 3 passages. The primary fibroblasts were cultivated in fibroblasts medium supplemented with 2% (v/v) of FBS and 1% (v/v) of fibroblast growth supplement. The monocytes were isolated from buffy coat and the peripheral blood mononuclear cells (PBMCs) were obtained after a centrifugation step (1200 g, 30 min with acceleration 5 and no brake). RosetteSep Human Monocyte Enrichment Cocktail (15 028, STEMCELL Technologies) reagent was used according to the manufacturer's instructions to separate the monocytes from the others PBMCs. Afterward, the content was diluted in PBS with 2% (v/v) of FBS, added in falcon tubes containing Histopaque‐1077 (10 771, Sigma) and centrifuged at 1200 g during 30 min with no acceleration and no brake. The monocytes were collected, washed 3 times in clean PBS and they were ready to assembly MCTS. The immortalized cells, A375 cells and HaCaT were cultured in DMEM supplemented with 10% (v/v) of FBS, 1% (v/v) of pen‐strep, and 1% (v/v) of non‐essential amino acids.

### Multicellular Tumoroids (MCTs) Formation

MCTs were formed using microtissue molds (3D Petri Dish, MicroTissues, Merk, USA) which were assembled using agarose 2% (w/v) in 0.9% NaCl (w/v). For mono‐culture it was adopted 5000 of A375 melanoma cells per spheroid, for double‐culture, triple‐ and quadruple‐culture it was used a ratio of 2:1, 2:1:1 and 2:1:1:1 of keratinocytes (isolated from foreign skin or HaCaT), fibroblasts (isolated from foreign skin or primary human dermal fibroblasts from biobank) and monocytes respectively. For all MCTs configuration it was maintained 5000 cells per spheroid. The condition called immune MCTs (iMCTs) is the quadruple‐culture, which was the condition with a combination of melanoma cells, keratinocytes, fibroblasts and monocytes. The cells suspension for each condition were added to the micro‐molds in 190 µL of DMEM and allowed to settle for 30 min, before adding complete DMEM to each well, covering the molds. The macrophage differentiation from human peripheral monocytes occurred spontaneously in situ in absence of colony‐stimulating factor or other exogenous factors. Medium was changed every 2 days and MCTs were incubated in a humidified atmosphere, at 37 °C, with 5% CO2.

### MCTs Characterization—Growth and Metabolic activity

The growth was evaluated during the time using a Zoe Microscope (Bio‐Rad Laboratories). Ten different MCTs of each condition, were randomly selected, and the acquired images were used for the growth analysis during time (Day 4, 7, and 10). High‐throughput imaging by Operetta High Content Imaging System was also adopted to evaluate important parameters to determine spheroids development and growth such as diameter (Equation (1)), perimeter (Equation (2)), sphericity (Equation (3)), solidity (Equation (4)), homogeneity (likelihood of adjacent pixels being equal) and convexity (Equation (5)).^[^ ^62^ ^]^

(1) $$Diameter=\frac{2\;area}{\pi }$$

where the area is usually determined by circumference area, and π = 3. 14 159 265. Perimeter is a measure that considers all the circumference, where r is the radius.

(2) Perimeter=2πr

Sphericity measures the degree to which an object approaches shape of a sphere.

(3) $$Sphericity=\frac{\pi \;Diameter}{Perimeter}$$

Solidity measures the density of an object, in this case taking into consideration the area or can be by pixels intensity.

(4) $$Solidity=\frac{Area}{Convex\;Area}$$

Convexity measures how much an object deviates from being 100% convex.

(5) $$Convexity=\frac{Convex\;Perimeter}{Perimeter}$$

In addition, at the same timepoints, five MCTs of each condition were harvested from the mold and the cells were dissociated using trypsin to count the number of total cells. Trypan Blue 0.4% solution (Gibco, Thermo Fisher Scientific) was used to mark the dead cells. The metabolic activity was determined using CellTiter‐Glo 3D cell viability assay reagent (Promega) following the manufacturer's instructions. This reagent was a luminescent cell viability assay, determining the number of viable cells based on quantitation of the ATP present there. It was pippeted 3 spheroids per well for a 96‐well plate in biological triplicate for each condition of MCTs, mono‐, double‐, triple‐, and quadruple‐culture, on the 4^th^, 7^th^, and 10^th^ in culture. In each well containing 3 spheroids in 100 µL of media, was added 100 µL of CellTiter‐Glo reagent and the plate was incubated during 30 min. After incubation the reagent content was transferred for a white 96‐well plate providing one well of space between the samples. The luminesce was determined by a Synergy microplate reader (BioTek).

### MCTs Characterization—Histology and Histochemistry

Paraffin‐embedded samples were sectioned into 3 µm sections (Leica RM2255 microtome) and were dewaxed in xylene, rehydrated in graded alcohol series of decreasing concentrations, and washed in distilled water. Afterward, samples were immersed in Gill's Hematoxylin solution (6 765 008, Lusoplex) for 3 min, dehydrated by immersing them in a graded alcohol series of increasing concentrations, followed by staining with alcoholic eosin (6 766 008, Lusoplex) for 1 min. Samples were washed in ethanol, immersed in xylene, mounted with Entellan mounting medium (107 961, Merck). Regarding histochemistry, sections were dewaxed and hydrated as mentioned before, stained by Masson Trichrome (Blue Collagen) (Epredia, Fisher Scientific) followed by dehydration and mounted with Entellan. Images were acquired using Brightfield Microscope Leica DM2000 LED.

### MCTs Characterization—Immunohistochemistry (IHC)

IHC assays were performed with anti‐S100 Monoclonal Antibody (1:100, clone 4C4.9, Catalog #MA5‐12969, Invitrogen, Rockford, UK) and Ki‐67 (1:500, clone SP6, Catalog #MA5‐14520, Invitrogen, Rockford, UK) in 4 um tissue sections. All immunohistochemistry protocol, dewaxing included, was performed automatically in a Ventana Discovery Ultra Platform (Roche Diagnostics, Tucson, USA) using CC1 as epitope recovery (ER). The downstream IHC protocol was performed as follows: MCT sections were blocked using UltraVision Protein Block (Epredia^©^) following an incubation step for 1 h at 37 °C with anti‐S100. Detection was performed with PoliviewPlus HRP (anti‐Mouse) over a 45 min incubation. Reactions were detected with 3,3′‐Diaminobenzidine (DAB) (Dako, Glostrup, Denmark) which converts the product of the reaction in a brown product. Tissues were then counterstained with HIGHDEF hematoxylin (Enzo, Farmingdale, USA), dehydrated and mounted with Entellan mounting medium (107 961, Merck). Positive and negative controls were included in every set of reactions for each antibody used. Images were acquired using Brightfield Microscope Leica DM2000 LED.

### MCTs Characterization—MCTs Ultrastructure

MCTs were removed from the agarose molds at day 7 of growth, washed in PBS twice to remove the media and fixed ON in 2% (w/v) glutaraldehyde solution in 0.1 m sodium cacodylate (pH 7.4). The spheroids were stained ON at 4 °C, in a 2%(v/v) osmium tetraoxide solution in 0.1 m sodium cacodylate. After staining, they were washed tree times per 10 min with 0.1 m cacodylate. The improve the microscopic visualization the iMCTS were transferred to 1% (v/v) uranyl acetate and incubated 1 h at 4 °C in the dark. Then, the iMCTS Spheroids were immobilized in Histogel and dehydrated in a gradient series of ethanol solutions (70%, 80%, 90%, and 100%) and finally in a propylene oxide solution for 10 min each. Thus, the iMCTS were included in EPON resin by gradual immersion of increasing series of propylene oxide to EPON (3:1, 1:1, 1:3 and 0:1) during 1 h each. After that, the inclusion in EPON resin was performed in a silicon mold. EPON polymerization was incubated at 60 °C for 48 h. Sections with 60 nm thickness were prepared using a diamond knife (Diatome, Hatfield, PA, USA) and were recovered to 200 mesh Cu‐grids. Staining of sections using 2% uranyl acetate (w/v) was performed before observation. The images were acquired at 80 kV in a Jeol JEM‐1400 transmission electron microscope (Japan) with a CCD digital camera Orious 1100 W (Tokyo, Japan).

### MCTs Characterization—Cellular Spatial Localization and Population Percentages

The cellular spatial localization and also populations percentages in the MCTs were determined using fluorescent labelled cells being the A375 labeled with CellTrace Far Red (Invitrogen, C34572; 630/661 nm), the keratinocytes labeled with CellTrace Yellow (Invitrogen, C34573; 532/561 nm), the fibroblasts labeled with Vybrant CFDA SE CellTrace and the monocytes labeled with CellTrace Violet (Invitrogen, C34557, 405⁄450 nm). The cells were labeled in suspension according to the manufacturer's protocol before the MCTs’ assembly. The MCTs were analyzed at day 7 in culture. The nuclei were stained with Hoechst 33 342 (MedChemExpress, HY‐15559) before analysis, with exception of quadruple‐culture to avoid overlapping of fluorescence signals. For spatial localization the 3D structures were observed in PerkinElmer Opera Phenix Microscope for high‐throughput acquisition. Since the MCTs presented strong production of ECM they were also observed in the THUNDER Imaging Systems (Leica) to clear view the details through computational clearing. The same procedure of labeling was made to produce MCTs for determining the population percentages, by flow cytometry. Eighty‐one (1 mold) MCTs of each condition were harvested from the microtissue molds to 15 mL falcon tubes and centrifuged for 5 min, at 1200 rpm, 4 °C. To dissociate the MCTs into single cells, all samples were incubated with 400 µL of Versene during 15 min at 37 °C. After that, they were centrifugated at 1500 rpm during 10 min, RT, followed by an incubation with 200 µL Accutase during 20 min at 37 °C. They were washed with PBS, fixed with 1% (v/v) PFA and analyzed in the BD FACSCanto II flow cytometer (BD Biosciences, USA). The compensation was made using cells from unstained MCTs.

### MCTs Characterization—Immunofluorescence

Paraffin‐embedded samples sectioned into 3 µm were dewaxed in xylene and rehydrated in a graded alcohol series of increasing concentrations. Slides were then incubated in sodium citrate buffer (0.01 m, pH 6) during 30 min, at 96 °C to allow antigen retrieval. Samples were permeabilized with 0.25% TritonX‐100 (Fisher Scientific) and blocked with 10% FBS (v/v) in PBS 1x during 1 h at RT. After permeabilization and blocking the slices were incubated with primary antibodies for HMB‐45, vimentin, fibronectin, EPCAM and CD68 in a wet chamber at 4 °C ON. After that, the slices received the secondary antibody, incubating during 1 h RT. Nuclei were stained with 4′,6‐diamidino‐2‐phenylindole (DAPI) (D4592, Sigma‐Aldrich, 0.5 µg mL^−1^) and the cytoskeleton were stained with Alexa Fluor 546 Phalloidin (A22283, Sigma‐Aldrich) diluted in diluted in 5% (v/v) FBS. The specifications of primary and secondary antibodies are listed in Table S2 (Supporting Information).

### Determination and Characterization of Immune Population

The immune population of the immune MCTs (quadruple‐culture) was characterized using flow cytometry. Eight‐one (1 mold) MCTs were harvested from the microtissue molds to 15 mL falcon tubes and centrifuged for 5 min, at 1200 rpm, 4 °C. To dissociate the MCTs into single cells, all samples were incubated with 400 µL of Versene during 15 min at 37 °C. After that, they were centrifugated at 1500 rpm during 10 min, RT, followed by an incubation with 200 µL Accutase during 20 min at 37 °C. They were washed with PBS and incubated in an antibody solution composed by anti‐CD14‐APC (1:100), anti‐CD86‐FITC (1:50) and anti‐CD163‐PE (1:25) during 30 min at 4 °C. Afterward, the samples were incubated with the APC‐Cy7‐Fixable Viability Dye eFluor 780 under the same conditions. Cells were fixed with 1% (v/v) PFA and analyzed in the BD FACSCanto II flow cytometer (BD Biosciences, USA). The compensation was made using unstained cells, died cells as a control of death, and also beads incubated with individual antibodies. It was considered CD14 positive cells the total immune population, CD68 for phenotype M1‐polarized macrophages and CD163 for M2‐polarized. The data were analyzed using FlowJo software which allow the generation os tSNE graphs. The FlowSOM were used to clustering by self‐organizing map. FlowSOM was used to distinguish cell populations in an unsupervised way. And X‐Shift algorithm was also applied to clusterization.

### Cytokines Secretion Levels

The MCTs in their day 10^th^ of culture were analyzed in terms of secretion of cytokines. Also, the same cytokines were determined in the supernatant of MCTs after 72 h of treatments. The cytokines were quantified by multiplex analysis which was performed using the Luminex 200 system (Luminex, Austin, TX, USA) by Eve Technologies Corp. (Calgary, Alberta). Forty‐eight markers were simultaneously measured in the samples using Eve Technologies' Human Cytokine Panel A 48‐Plex Discovery Assay (Millipore Sigma, Burlington, Massachusetts, USA) according to the manufacturer's protocol. The 48‐plex consisted of sCD40L, EGF, Eotaxin, FGF‐2, FLT‐3 Ligand, Fractalkine, G‐CSF, GM‐CSF, GROα, IFN‐α2, IFN‐γ, IL‐1α, IL‐1β, IL‐1RA, IL‐2, IL‐3, IL‐4, IL‐5, IL‐6, IL‐7, IL‐8, IL‐9, IL‐10, IL‐12(p40), IL‐12(p70), IL‐13, IL‐15, IL‐17A, IL‐17E/IL‐25, IL‐17F, IL‐18, IL‐22, IL‐27, IP‐10, MCP‐1, MCP‐3, M‐CSF, MDC, MIG/CXCL9, MIP‐1α, MIP‐1β, PDGF‐AA, PDGF‐AB/BB, RANTES, TGFα, TNF‐α, TNF‐β, and VEGF‐A. Assay sensitivities of these markers range from 0.14 – 50.78 pg mL^−1^ for the 48‐plex. Individual analyte sensitivity values are available in the MILLIPLEX MAP protocol. The data were analyzed using Python 3.9 with Pandas package for pre‐processing data and Seaborn for generating the heatmaps and Clustermaps. The plots were created from normalized data as z‐score.

### Screening of Therapies

The screening of therapies was based on the evaluation of MCTs some parameters such as diameter (size) and area changes, detaching cells, decrease of metabolic activity and MCT architecture after treatments. At day 7 of culture the MCTs were incubated with treatments and the parameters cited were determined in 24, 48, and 72 h within treatment exposure. The treatments were: Dacarbazine (DAC) (50, 100, 500, 1000 µm), a gold standard chemotherapy for melanoma; Vemurafenib (VEM) (5, 15, 50, 150, 200 µm), the first BRAF inhibitor used to treat advanced malignant melanoma; Temozolomide (TMZ), (50, 100, 500, 1000 µm), the first‐choice drug for brain melanoma metastasis; and Paclitaxel (PTX), (50, 100, 500, 1000 µm), usually used in combinatory chemotherapy regimens for melanoma. The diameters were determined using Operetta High Content Imaging System (PerkinElmer) for high‐throughput acquisition adopting a 5 × 5 matrix in the center of 9 × 9 molds in 12‐well plates. The acquisition captured at least 15 spheroids in each mold, being triplicate for each drug's dosage. The determination of detaching cells was determined by ImageJ NIH using Zoe microscope images, and the metabolic activity was determined by CellTiter‐Glo 3D as well as in the characterization. The supernatant of microtissue‐molds was analyzed regarding cytokines secretion by the same multiplex assay mentioned before. The data were analyzed using Python 3.9 with Pandas package for pre‐processing data and Seaborn for generating the heatmaps, Clustermaps and correlation heatmaps. The Spearman network correlation plots were built using Pandas and NetworkX. The plots were created from normalized data as z‐score.

### Statistical Analysis

Statistical analysis was performed using Graph‐Pad Prism 8 software. The one‐way ANOVA test followed by Tukey's multiple comparison. Kruskal–Wallis test followed by Dunn's test were performed for non‐parametric data. A value of *P* ≤ 0.05 was considered statistically significant, in a 95% confidence interval.

## Conflict of Interest

The authors declare no conflict of interest.

## Author Contributions

J.V. and B.S. performed conceptualization. J.V., S.C., and S.D. performed methodology and investigation. J.V. and C.L.P. performed data curation. J.V. performed wrote the original draft. J.V. and B.S. performed funding Acquisition. B.S. provided resources and performed supervision. All authors wrote, reviewed, and edited the draft.

## Supporting information



Supporting Information

## Data Availability

The data that support the findings of this study are available from the corresponding author upon reasonable request.
