# Supplementary material for: Patient‐Derived Melanoma Immune‐Tumoroids as a Platform for Precise High throughput Drug Screening
Source: Adv Sci (Weinh). 2024 Oct 30;11(48):2408707. doi: 10.1002/advs.202408707 (PMC11672280; doi:10.1002/advs.202408707)
Supplement: Supplementary file 1 — Supporting Information [file ADVS-11-2408707-s001.docx]

**Supplementary Material**

**
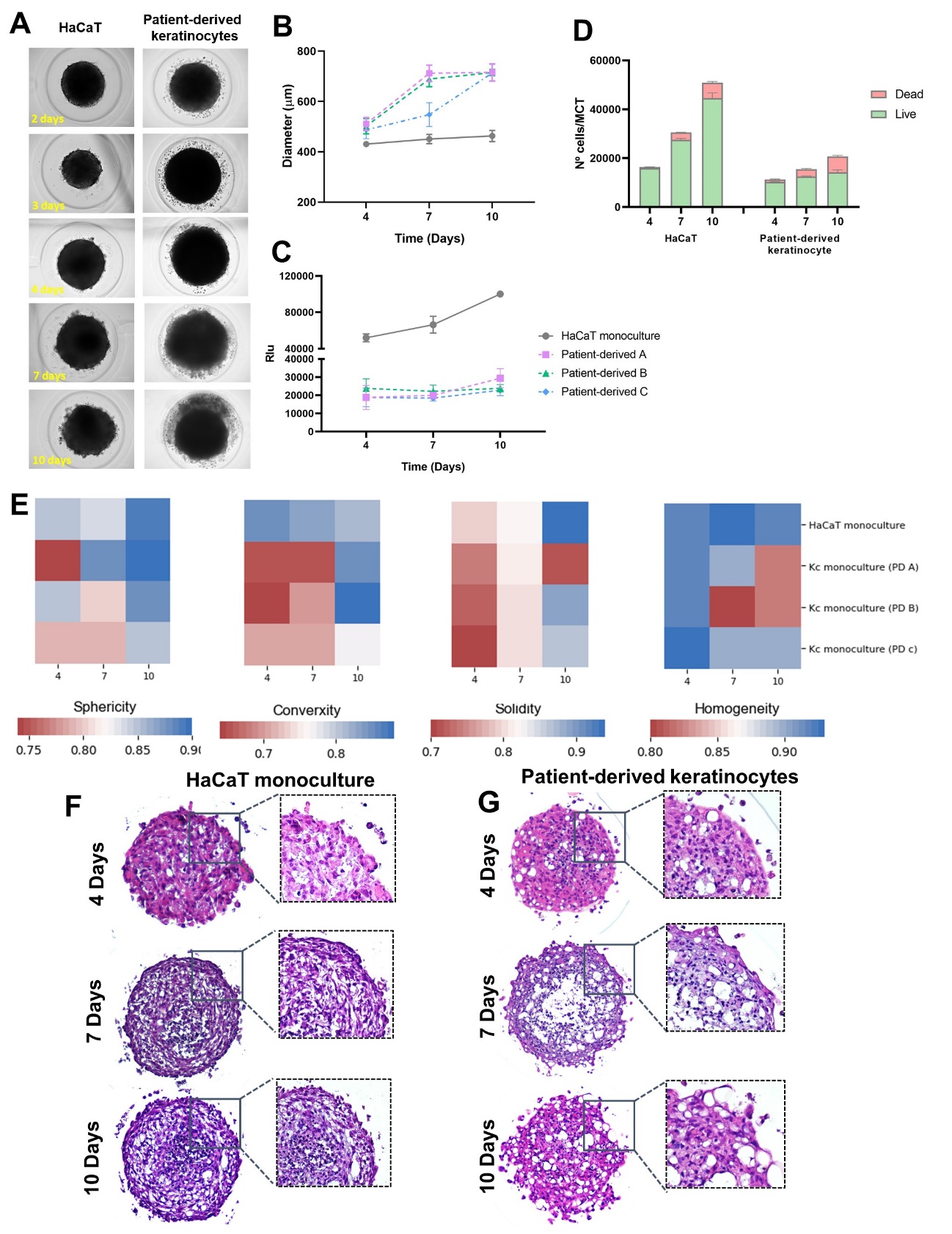
**

**Figure S1:** Monoculture spheroids of keratinocytes – immortalized and patient-derived, as control. (A) They were monitored using Zoe microscope and the media was changed every two days. (B) The diameters of MCTs were determined using ImageJ (NIH) software and OrganoSeg software. (C) The metabolic activity was measured by ATP (CellTiter-Glo^®^) and (D) the MCTs dissociation determine the number of cells in each MCTs and the number of them alive and dead. (E) Though Operetta^®^ High Content Imaging was possible to determine crucial parameters such as sphericity, convexity, solidity and, homogeneity. (F) Immortalized and (G) patient-derived spheroids architecture observed by hematoxylin-Eosin histological analysis. Data are represented as mean ± SD, n=10.

**
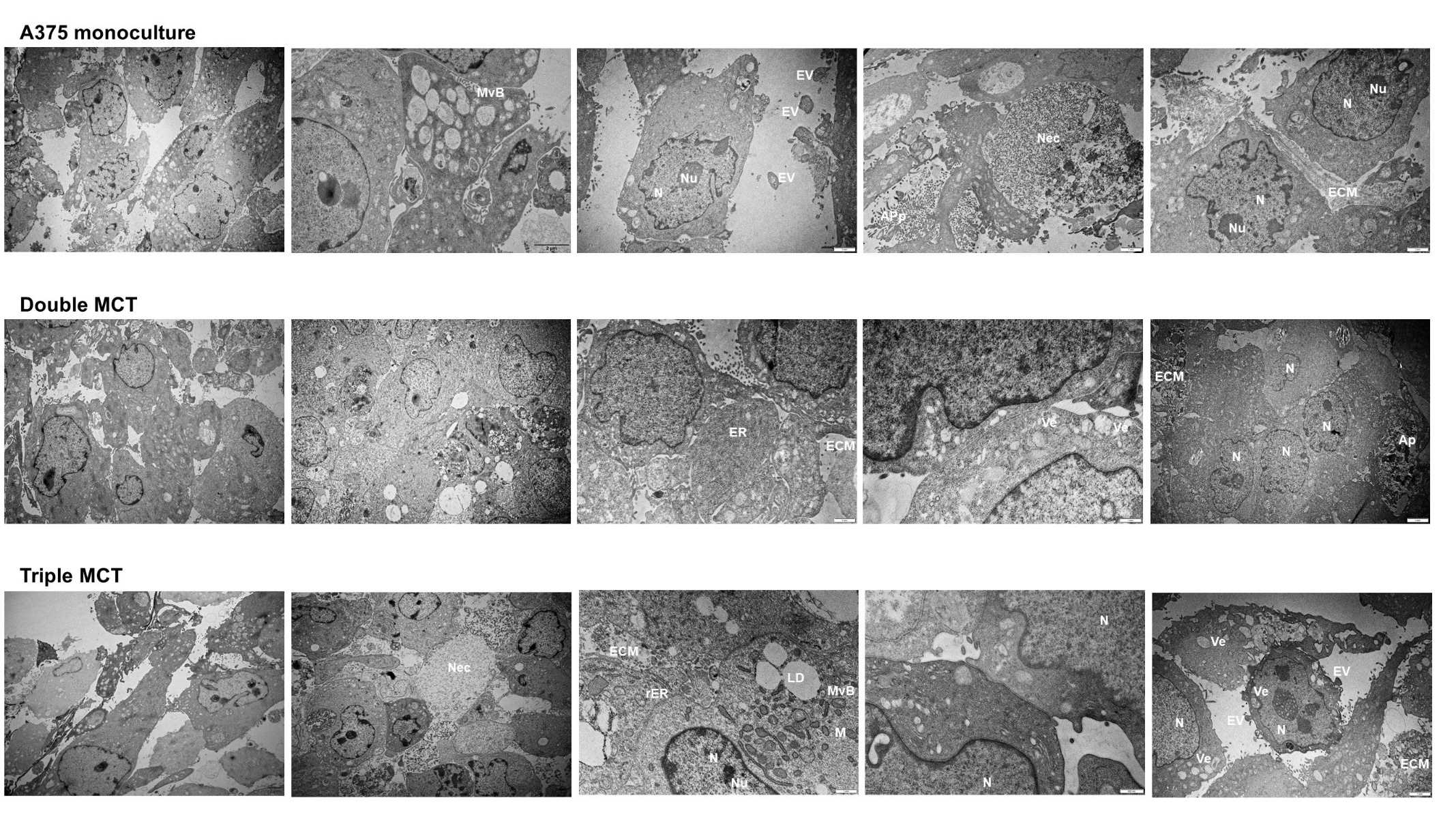
**

**Figure S2:** Transmission electron microscopy (TEM) ultrastructure imaging. Images showed cell-cell interactions and accumulation of ECM. Images were obtained with 12k, 15k and 35k magnification. These are representative images and, they were collected since the border to the center crossing to the opposite border. N = nuclei, Nu = nucleolus, ER = endoplasmic reticulum, rER = rough endoplasmic reticulum, M = mithocondria, EV = extracellular vesicles, Ve = vesicles, MvB = multivesicular bodies, App = apoptotic podies, Ap = apoptosis, Nec = necrosis, ECM = extracellular matrix, Tj = tight junctions, KRT = Keratin, LD = Lipid droplets. See also Figure S2 for TEM images of monoculture, double and triple MCTs.

**
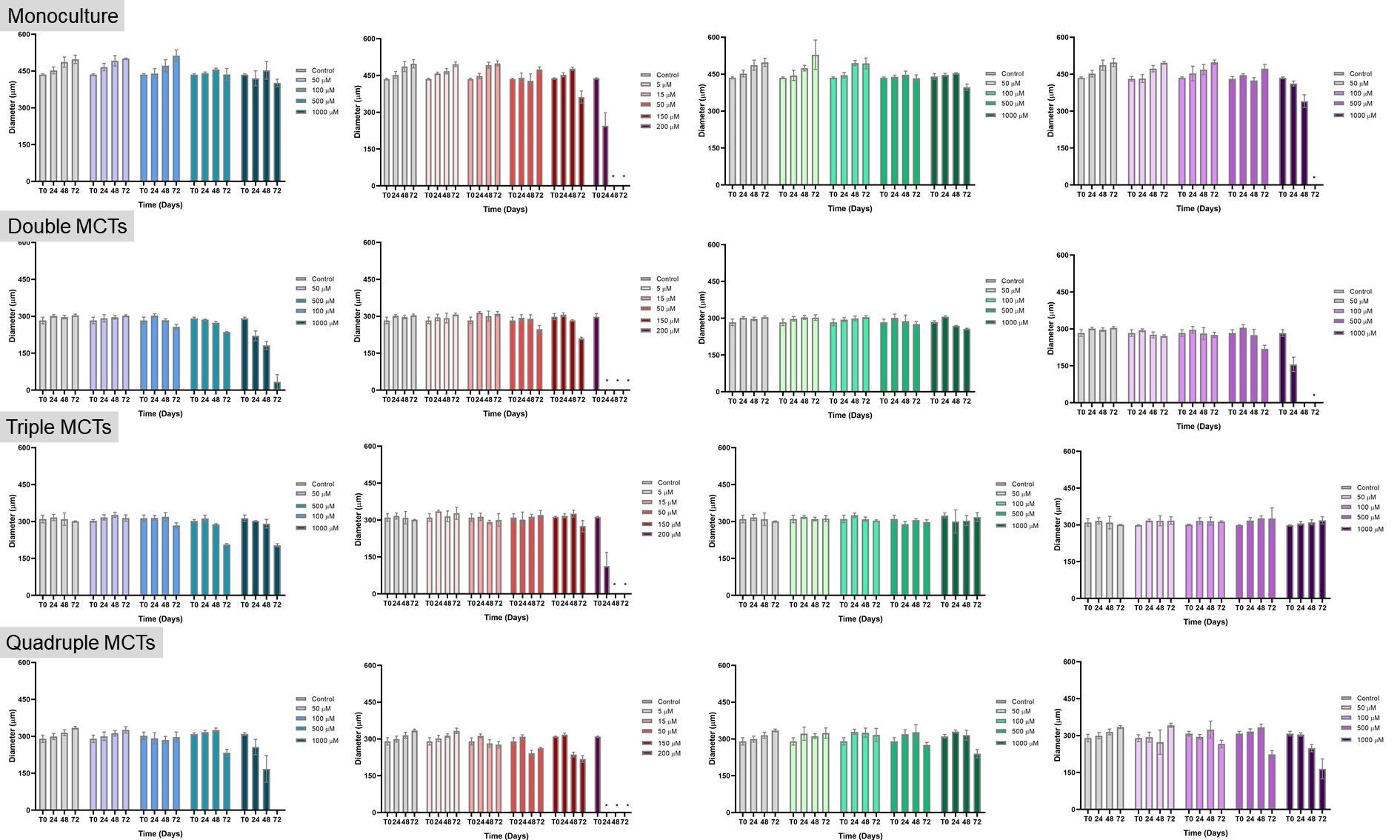
**

**Figure S3:** Drug screening performed in MCTs to determine the optimal dosage to be used in the experiments of cytokines secretion. Blue (first), DTIC. Red (second), VEM. Green (third), PTX; and purple (fourth), TMZ. * Means – it was no possible to determine diameter since the MCTs structured was lost (disaggregate or occurred cell death. Data are represented as mean ± SD, n=10.

**
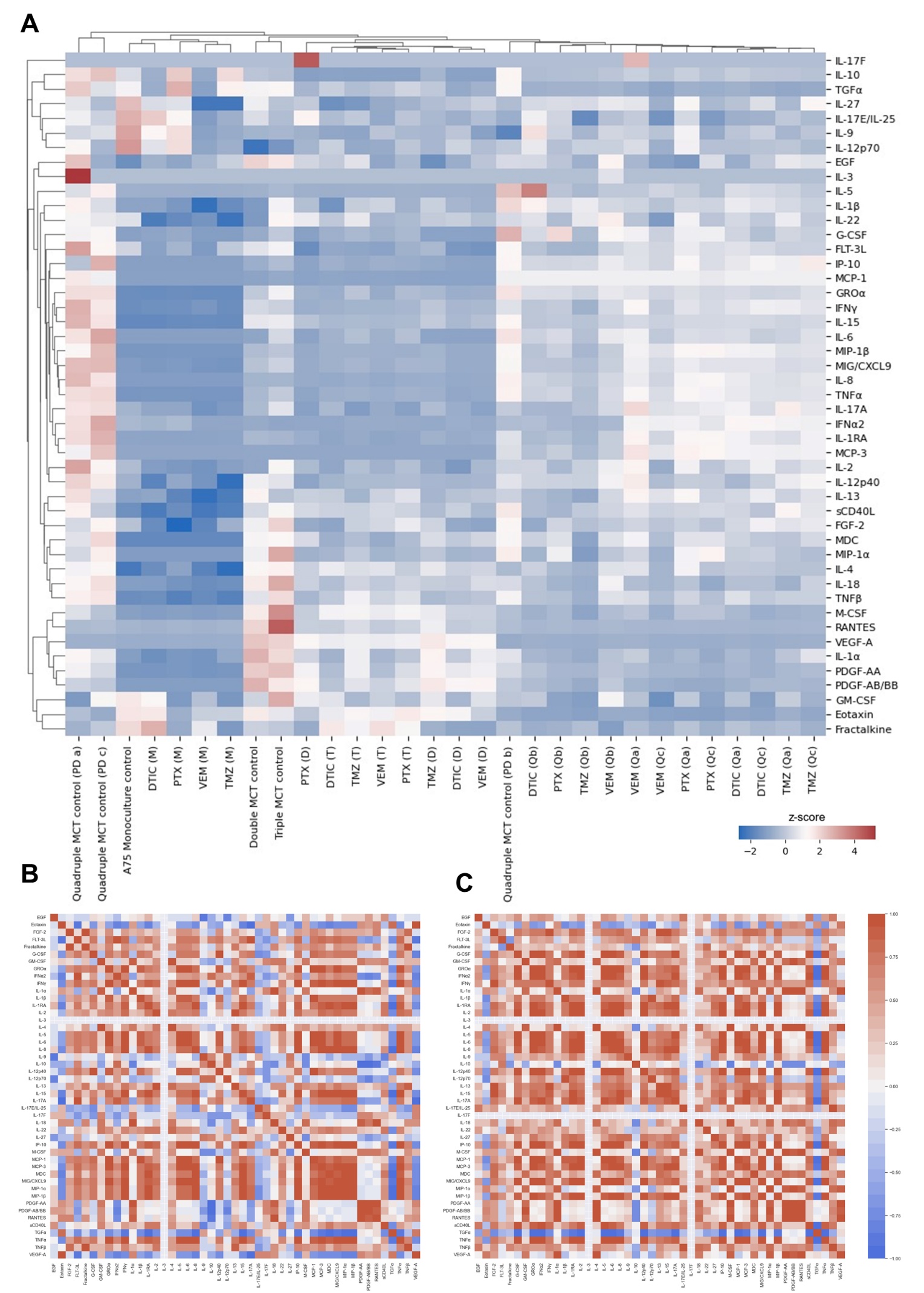
**

**Figure S4:** Cytokines panel (Human Cytokine Panel A 48-Plex Discovery Assay®) Heatmaps of MCTs controls and after each treatment with the drugs dacarbazine (DTIC) at 500 µM, Vemurafenib (VEM) at 150 µM, Paclitaxel (PTX) at 1000 µM and Temozolomide (TMZ) at 500 µM. The drugs concentrations were pre-determined among four, and stablished as the optimal for all MCTs. (A) Cluster Map and Correlation Heatmap of (B) PTX and (B) TMZ. The plots were created from normalized data as z-score.

**
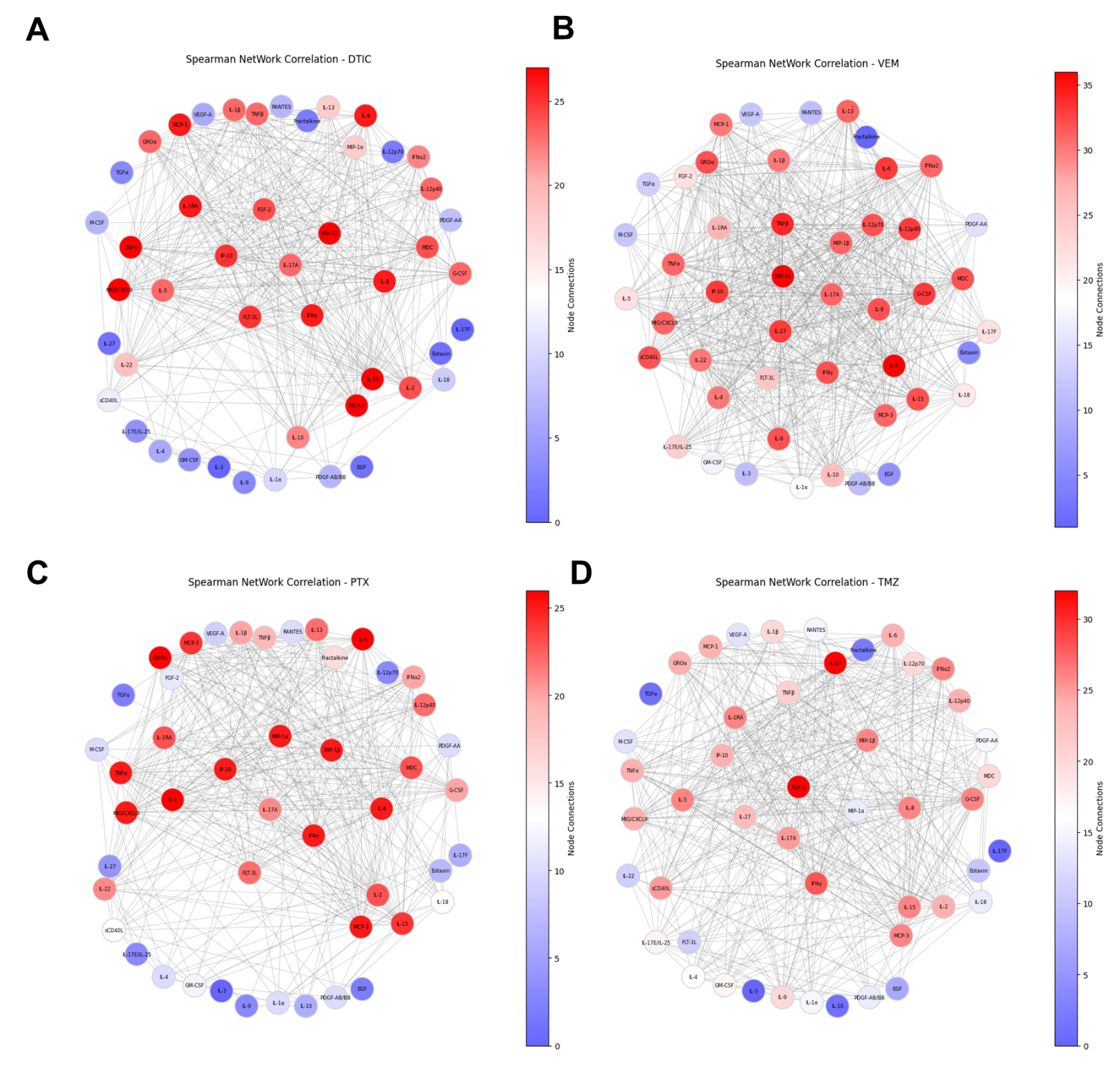
**

**Figure S5:** Cytokines Spearman Network Correlation for each treatment (A) DTIC, (B) VEM, (C) PTX and (D) TMZ after 72 hours of MCTs’ treatments exposure. Spearman network correlation plots were built using Pandas and NetworkX. The plots were created from normalized data as z-score.

**Table S1.** Summarization of the main cytokines involved with each MCT and the readout related to the combination of them regarding important feature that a MCT could present to be related with *in vivo* tumors.

| MCTs | Main Cytokines group involved | Main role | Feature generated | | | | |
| --- | --- | --- | --- | --- | --- | --- | --- |
|  |  |  | ***Proliferation*** | ***Invasiveness and Migration*** | ***Cell Death*** | ***Tratment Resistance*** | ***Complexity*** |
| Monoculture | IL-27, IL-12p70 | Anti-tumor, immune modulation/activation | Moderate | Limited | Higher | Lower | Low |
|  | IL-17E/IL-25, IL-9 | Immune response, inflammation, cell growth, survival |  |  |  |  |  |
|  | Fractalkine, Eotaxin | Chemotaxis, immune cell recruitment, inflammation |  |  |  |  |  |
| Double | VEGF-A, PDGF-AB/BB, EGF | Angiogenesis, cell proliferation, survival | High | Increased | Reduced | Moderate | Moderate |
|  | RANTES, IL-1α | Chemotaxis, immune cell recruitment, inflammation |  |  |  |  |  |
|  | Eotaxin | Eosinophil recruitment, inflammation |  |  |  |  |  |
| Triple | VEGF-A, PDGF-AB/BB, PDGF-AA | Angiogenesis, cell proliferation, survival | Very high | Significantly increased | Reduced | Higher | High |
|  | TNF-β, IL-18 | Inflammation, cell survival, proliferation |  |  |  |  |  |
|  | RANTES, MIP-1α | Chemotaxis, immune cell recruitment |  |  |  |  |  |
|  | M-CSF, GM-CSF | Monocyte differentiation, granulocyte/macrophage activation |  |  |  |  |  |
|  | IL-22, IL-4 | Inflammation, cell survival |  |  |  |  |  |
|  | FGF-2 | Cell growth, angiogenesis |  |  |  |  |  |
| Quadruple | TNF-α, TGF-α | Inflammation, immune modulation, cell proliferation, survival | Extremely high | Maximum | Minimaly reduced | Very high | Very high |
|  | MIP-1 β, MIG, MCP-3, MCP-1 | Chemotaxis, immune cell recruitment, immune surveillance |  |  |  |  |  |
|  | EGF | Cell proliferation, survival, migration |  |  |  |  |  |
|  | IP-10, IL-17A, IFN-α 2, IFN-γ | Immune cell recruitment, inflammation, immune activation, anti-tumor response |  |  |  |  |  |
|  | IL-15, IL-3, IL-2 | Intense immune activation |  |  |  |  |  |
|  | IL-13, IL-12p40, IL-10 | Immune modulation, anti-inflammatory |  |  |  |  |  |
|  | IL-8 | Chemotaxis, angiogenesis |  |  |  |  |  |
|  | IL-5 | Immune modulation |  |  |  |  |  |

**Table S2.** List of antibodies and staining used for Immunofluorescence imaging of MCTs.

| Specification | Target | Supplier | Reference | Host | Target species | Dilution used |
| --- | --- | --- | --- | --- | --- | --- |
| Primary | EpCAM | Invitrogen | MA1-10195 | Mouse | Human | 1:200 |
| Primary | HMB-45 | Invitrogen | MA1-34759 | Mouse | Human | 1:250 |
| Primary | Fibronectin | Sigma Aldrich | F3648 | Rabbit | Human | 1:400 |
| Primary | CD68 | Dako | M0876 | Mouse | Human | 1:100 |
| Primary | Vimentin | Santa Cruz Biotechnology | sc-6260 | Mouse | Mouse, rat, human | 1:200 |
| Secondary | Alexa Fluor™ 647 | Invitrogen | A-21235 | Goat | Mouse | 1:500 |
| Secondary | Alexa Fluor™ 488 | Invitrogen | A-11070 | Goat | Rabbit | 1:500 |
| Staining | DAPI | Merck | D9542 | - | - | 0.5 µg/mL |
| Staining | Phalloidin (Alexa Fluor™ 546) | ThermoFisher | A22283 | - | - | 1:2000 |
